# Current Perspectives on Nucleus Pulposus Fibrosis in Disc Degeneration and Repair

**DOI:** 10.3390/ijms23126612

**Published:** 2022-06-14

**Authors:** Yi Sun, Minmin Lyu, Qiuji Lu, Kenneth Cheung, Victor Leung

**Affiliations:** Department of Orthopaedics and Traumatology, The University of Hong Kong, Hong Kong SAR, China; ericsunhku@gmail.com (Y.S.); lvmm@hku-szh.org (M.L.); u3571660@hku.hk (Q.L.)

**Keywords:** nucleus pulposus, fibrosis, intervertebral disc degeneration, TGF-β1, myofibroblast

## Abstract

A growing body of evidence in humans and animal models indicates an association between intervertebral disc degeneration (IDD) and increased fibrotic elements in the nucleus pulposus (NP). These include enhanced matrix turnover along with the abnormal deposition of collagens and other fibrous matrices, the emergence of fibrosis effector cells, such as macrophages and active fibroblasts, and the upregulation of the fibroinflammatory factors TGF-β1 and IL-1/-13. Studies have suggested a role for NP cells in fibroblastic differentiation through the TGF-βR1-Smad2/3 pathway, inflammatory activation and mechanosensing machineries. Moreover, NP fibrosis is linked to abnormal MMP activity, consistent with the role of matrix proteases in regulating tissue fibrosis. MMP-2 and MMP-12 are the two main profibrogenic markers of myofibroblastic NP cells. This review revisits studies in the literature relevant to NP fibrosis in an attempt to stratify its biochemical features and the molecular identity of fibroblastic cells in the context of IDD. Given the role of fibrosis in tissue healing and diseases, the perspective may provide new insights into the pathomechanism of IDD and its management.

## 1. Introduction

The intervertebral disc (IVD) is the largest avascular, cartilaginous tissue in the human body. It is composed of a colloidal core, the nucleus pulposus (NP) and outer surrounding layers of annulus fibrosus (AF), sandwiched between two cartilaginous endplates. The mature NP in humans consists of a proteoglycan- and collagen II-rich extracellular matrix (ECM). Under compressive loading, the NP exerts circumferential tensile stress on the collagen I-rich AF lamellae structure. Such compartmental organization supports the capacity of the IVD to absorb shock while enabling the motion of spinal segments. IVDs undergo progressive degeneration in aging [1], characterized by inflammation-driven ECM degradation, altered growth factor activity and anabolic events, and the replacement of native disc cells with a heterogenous cell population [2,3,4,5,6,7]. The loss of ECM integrity in the NP results in a loss of water-retaining and load-bearing capacity. These alterations translate into mechanical dysfunction, ultimately leading to disc narrowing, bulging or herniation, causing nerve compression or agitation and back pain.

Fibrosis is commonly the pathological outcome of many chronic inflammatory diseases, including idiopathic pulmonary fibrosis, liver cirrhosis, left-ventricular hypertrophy and rheumatoid arthritis, to name just a few. It is characterized by a massive accumulation of fibrotic matrix (e.g., collagens and fibronectin) within focal inflamed or damaged tissues resulting from persistent myofibroblast activity [8]. Recent studies have demonstrated the abnormal deposition of fibrotic proteins in the ECM and the existence of fibroblastic NP cells during IVD degeneration (IDD), suggesting a fibrosis-like process. This phenomenon has attracted increasing interest, as it provides a clue to the cause of hardening and scarring of NP and consequent changes in IVD mechanics. That having been said, ECM-producing myofibroblasts also play a critical role in wound healing and therefore fibrosis might provide a temporary or compensatory reparative mechanism in IDD. Understanding NP fibrosis may not only shed new light on the pathomechanism of IDD but also be harnessed to discover new IDD-modifying therapeutics.

## 2. Matrices in NP Fibrosis

### 2.1. Proteoglycans

NP is rich in proteoglycans which contain highly sulphated glycosaminoglycan (GAG) side chains, mostly in the form of chondroitin sulfate (CS), keratin sulfate, heparin sulfate and hyaluronic acid (HA). GAGs are negatively charged (except for HA) and hydrophilic, and are responsible for viscoelastic properties of tissues through interaction with other matrices. Beyond their function in matrix scaffolding, GAGs possess morphogenic and homeostatic bioactivities by binding with and deploying various growth factors (e.g., TGF-β [9,10] and FGF [11,12]), cytokines (e.g., IL-10 [13] and CCL-5 [14]) and other distinct signaling molecules (e.g., STING [15]) [16]. GAGs are therefore important regulators of tissue fibrosis. For example, hyaluronic acid can induce myofibroblast generation and fibrosis via CD44–EGFR complex formation, engaging ERK and CAM kinase activation [17]. Chondroitin sulfate can prevent peritoneal fibrosis in mice by suppressing NF-κB activation [18] and is involved in cardiac fibrosis via direct binding with TNF-α [19].

IDD is associated with a reduction in GAG content [20] along with fibrosis-like changes in NP containment [21]. CS, particularly chondroitin-4 sulfate, is the major GAG expressed by NP cells and its levels are decreased in IDD [22,23]. One major family of GAG-containing matrix proteins are small leucin-rich proteoglycans (SLRPs), which are crucial signal transducers and receptors. SLRPs have received attention in the IVD field in view of their roles in development, matrix functionality and tissue remodeling [24]. Enrichment of decorin, biglycan, lumican, fibromodulin, clusterin, fibronectin, chondroadherin, cartilage intermediate layer protein (CILP), proline/arginine-rich end leucine-rich repeat protein (Prelp) and cartilage oligomeric matrix protein (Comp), to name the most prominent, have been reported in the NPs of degenerative and aged human IVDs [21] and animal models [25,26]. Among the SLRPs, biglycan, decorin, fibromodulin, versican, fibronectin, Prelp and Comp are known to enhance collagen fibril formation. In line with their role in fibrillogenesis, the upregulation of biglycan [27] and decorin [28] has been linked to tissue fibrosis. In the context of IDD, decorin was found to be increased in degenerative NPs [22] and could stimulate pro-inflammatory and chemokine production in rat coccygeal IVDs [29]. Biglycan expression was found in fetal NPs [30] and increased with aging [21] and the degeneration of discs [22]. However, biglycan deficiency could also accelerate the IDD process [31], implying its pleiotropic role in homeostasis and the degenerative process. Lumican facilitates collagen fibril fusion and may accelerate joint fibrosis through TGF-β activation in joint synovial fibroblast [32]. Increase in fibronectin and its fragments has been linked to IDD and its fibrotic-like events [21,25,26]. While the extra domain A isoform of fibronectin (FN-EDA) can act as a damage-associated molecular pattern fragment to elicit sterile inflammation, as in immune cells and dermal fibroblasts [10], FN-EDA could not be detected in IVDs [21,33]. This implies that fibronectin may partake in IDD and NP fibrosis via other functions, such as collagen fibril interaction. Clusterin, which attenuated hepatic fibrosis through the downregulation of Smad3 in hepatic stellate cells [34], was found to be elevated in osteoarthritic cartilage [35] and IDD [21]. IDD is also associated with the expression of periostin [36,37], which can bind directly to collagen I and integrin αvβ3 and promote macrophage recruitment and p38 activation in fibrosis of the kidneys [38] and lungs [39]. CILP can modulate ECM metabolism jointly with TGF-β1 or IGF-1 [40] and has been associated with IDD [40,41] and NP fibrosis [21]. CILP-targeting microRNA miR-330-5p was recently shown to reduce cellular senescence and increase collagen I production in NP cells [41]. Perlecan, or heparan sulphate proteoglycan 2, was found to be expressed in intervertebral disc chondrons [42]. It is known to play an important role in fibrogenesis across different tissue types by coordinating collagen fibrillogenesis in a temporal and dynamic fashion [12]. Perlecan can also interact with FGF family members and regulate their activities [12]. Syndecan 4 is a cell-surface heparan sulphate proteoglycan involved in matrix degradation via control of ADAMTS-5 function and MMP-3 expression in disc cells [43] and promotes IDD via the activation of JNK/p53 [44]. Syndecan 4 was found to inhibit lung and heart fibrosis by limiting myofibroblast activity and attenuating the TGF-β pathway [45,46]. Whether Perlecan and Syndecan 4 are implicated in NP fibrosis is still an open question. Taken together, the enrichment of SLRPs and their fragments in IDD may potentially facilitate profibrotic activities in discs.

### 2.2. Collagens in NP Fibrosis

Collagens are the major players in tissue fibrosis, and alteration of their molecular composition and spatial distribution in fibrosis has been broadly studied [47]. Polymorphisms in collagen-encoding genes have been associated with IDD, including *COL1A1*, *COL2A1*, *COL9A2*, *COL9A3*, *COL11A1* and *COL11A2* [48]. Most of these collagens are linked to the regulation of fibril assembly and cell adhesion, and their dysregulation may lead to fibrosis. Collagen II is predominantly expressed in healthy NPs to support matrix meshwork, rendering NP mechanical strength and elasticity. Collagen II expression in the NP decreases with aging and degeneration [21]. Various animal model studies suggest that early IDD involves a transient increase in collagen II, changing gelatinous NP into a cartilaginous entity before further remodeling into fibrous tissue [25,49], characterized by an increase in collagen I. Accumulation of collagen I has been evidenced in human IDD [6,21,50], animal models of IDD induced by aging [6,21,25,49,50,51], overloading [52] or injury [6,26,36,49,53,54,55,56,57,58,59], as well as NP cells under stress [36,50] or treatment of TGF-β [59,60]. Collagen III and collagen I are primary ECM components produced by myofibroblasts that are proposed to increase tissue stiffness and tensile force [61] and decrease tissue compressive strength. In IVDs, collagen III is lowly expressed in the boundary zone between NPs and AFs. Its expression level can be significantly increased by TGF-β1 in degenerative NP cells [60]. Col6a1, Col6a2, Col12a1 and Col15a1 are minor collagens in IVDs [21], and their expression levels are increased in SM/J mice, which present IDD with fibrotic-like NPs during aging [25]. Heat shock protein 47 (HSP47), a chaperone for intracellular collagen assembly and processing, is also linked to NP fibrosis [6,58].

Abnormal collagen accumulation and fibril assembly has been detected in IDD. Collagen fibrils from NPs in a rabbit disc injury model showed less uniformity and significantly larger diameters when compared with normal NPs [58]. The formation of thicker collagen fibrils might be associated with excessive mechanical stress or TGF-β1 signaling [62]. However, the analysis of human IVD samples indicated smaller fibril diameters in degenerative NPs ranging from 50.59 nm to 64.76 nm versus 60.01 nm to 77.63 nm in non-degenerative NPs [21]. This is consistent with findings for age-related IDD in Tnmd-knockout mice [63], suggesting that dysregulated collagen fibrillogenesis may be involved in the late stage of IDD. The disparity in fibril changes might be due to differences in the species and natures of IDD. Thinner and disorganized collagen I fibers are also detected in fibrotic alteration of AF [64]. He et al. recently reported that collagen fibrils were thicker in aging than in punctured IVDs [7]. However, age-matched controls were not examined. Tissue porosity tends to increase in degenerative NPs (8.1~16.2% vs. 6.36~14.97%) [21,58], which likely increases cell interconnectivity [65] and nerve ingrowth [66]. Intriguingly, the size of collagen fibrils can modify the morphology and phenotype of AF-derived stem cells in vitro, implying that a dysregulated collagen meshwork can impact disc cell differentiation and function [67]. How collagen fibrillogenesis influences the phenotype of NP cells warrants further investigation.

## 3. Cell Composition in NP Fibrosis

The increased heterogeneity of NP cells in IDD has been widely reported. Large vacuolated notochordal NP cells normally vanish in adult humans and are replaced by small, rounded chondrocyte-like cells. Mesenchymal stromal cell (MSC)-like progenitors in the NP [5] and the AF [68] were found to be reduced in aging and IDD. In addition to a change in local cell entities, reports have suggested the infiltration of extrinsic cells. Accumulation of macrophage- (CCR7^+^, CD163^+^ [4] and CD68^+^ [69]) and myofibroblast-like (α-SMA^+^) [6,64,70] cells were documented in degenerative NP and AF. Additionally, the migration of bone marrow-derived MSCs into NPs were noted in a mouse tail-looping IDD model [71]. How these cells interact and participate in NP fibrosis is an issue worth addressing.

While macrophage-like cells have been detected in degenerative IVD, their source and role in NP fibrosis is still unclear. Tissue fibrosis is driven by inflammation. During the inflammatory response, M1 macrophages secrete pro-inflammatory cytokines, such as IL-1 and TNF-α, at the early stage and M2 macrophages produce anti-inflammatory factors, such as IL-10 [72], at the later stage. The latter stimulates the activation of myofibroblasts to aid tissue repair and wound healing. M1 macrophages activate NF-κB and promote myofibroblast transition of MSCs [73] and epithelial cells [74]. Activated macrophages also produce pro-fibrogenic factors (e.g., TGF-β1) to enhance the proliferation and activation of collagen-producing fibroblasts. Interestingly, inflammatory macrophages were shown to be capable of undergoing myofibroblast transition in renal fibrosis [75]. The finding of colocalization of the macrophage marker MMP-12 with α-SMA in the NP of induced IDD appears to support such a transition [6,50].

Myofibroblasts are active, contractile forms of fibroblasts and are regarded as primary effector cell types in tissue fibrosis. Myofibroblasts possess a high capacity of ECM production and are characterized by cytoskeletal features of contractile smooth muscle cells and expression of the smooth muscle actin α-SMA as a marker [76]. GLI-1 and FAP-α are hallmarks of myofibroblast precursors [77]. A subset of fibroblasts expressing FSP-1 were identified in the heart, kidneys and skin [78], while an FSP-1 expressing myeloid-monocytic cell lineage was found to be involved in liver injury [79]. Myofibroblasts could be derived from mesenchymal stromal cells [80], epithelial–mesenchymal transition (EMT, [81]), circulating fibrocytes [82], pericytes [83] and macrophages [6]. In both cultured disc cells and a mouse annulus puncture-induced IDD model, treatment with bleomycin, a commonly used fibrosis-inducing agent, resulted in the expression of fibroblastic markers [26]. This may imply the intrinsic capacity of NP cells for myofibroblastic transition. NP cell lineage tracing in an induced IDD model also suggested that resident notochord-derived NP cells can be an origin of myofibroblasts [49]. In that study, however, not all myofibroblastic cells could be traced to local NP cells, suggesting additional origins of the fibroblastic cells. For instance, focal upregulation of myofibroblasts near infiltrating blood vessels has been demonstrated at the lesion site in an annulus incision model, suggesting that myofibroblasts might also be recruited from circulation through local inflammatory stimuli [70]. On the other hand, Chen et al. reported that extrinsic fibroblasts could induce NP cells to acquire a fibroblastic phenotype expressing FSP-1 and collagen I, indicating the possibility of a paracrine inductive mechanism [55]. The roots and routes of fibroblastic and myofibroblastic cell emergence and their regulatory mechanisms remain to be addressed.

## 4. Regulation of NP Fibrosis

### 4.1. Growth Factors

TGF-β is extensively implicated in the pathogenesis of fibrosis. Rapid collagen deposition by TGF-β stimulation is thought to promote tissue strength in the fibrosis of multiple organs [47]. The main ligand invoking fibrosis appears to be TGF-β1 [54]. TGF-β signaling is thought to play a role in the repair of IVDs by promoting matrix synthesis and reducing catabolism and inflammatory activities [3]. However, excessive and persistent TGF-β activation can be detrimental, and inhibition of its aberrant activity can retard IDD [3,59,84,85,86]. Aberrant mechanical loading can cause cartilage hypertrophy in EP [85] and loss of notochordal cell vacuoles in NP [86] and hence IDD due to excessive activation of TGF-β signaling. Inactivation of TGF-β via curcumin [84], ALK-5 inhibitor [85] and NR4A1 (orphan nuclear receptor 4A1 [59]) could ameliorate IDD. The precise mechanisms regarding the different roles of TGF-β in the context of NP fibrosis await definition.

Besides its pro-chondrogenic/protective effects, TGF-β can induce unchecked myofibroblast activity. A number of studies have implicated TGF-β in NP fibrosis and the myofibroblastic differentiation of NP cells. TGF-β1 treatment could induce α-SMA and collagen I expression in human NP cells [59]. Increase of collagen I and III by IL-1β could be aggravated by TGF-β1 via regulating angiopoietin-like protein 2 (ANGPTL2) expression [60]. Fibroblastic phenotype induction of NP cells by co-culture with fibroblasts is dependent on Smad2/3 activation [55]. Increased FSP-1, collagen I and fibronectin in human NP cells due to bleomycin treatment also involves the TGF-β-Smad2/3 pathway [26]. Loss of NR4A1 can perpetuate TGF-β activity and pathological tissue fibrosis in many organs. NR4A1 was suggested to bind with SP1 to repress TGF-β target genes in rat injury-induced NP fibrosis [59]. Smad-mediated signaling can crosstalk with non-Smad pathways, such as MAPKs [87] and Wnt/β-catenin signaling, to regulate fibrotic response [88]. In IVDs, altered Wnt or MAPK signaling was found to promote cellular senescence, apoptosis, autophagy and inflammatory responses, thereby accelerating the degeneration process [89,90]. Whether these non-Smad related pathways are involved in NP fibrosis and, if so, how the different pathways coordinate fibrotic progression are questions that await examination. Sox9 is a well-known master transcription factor for chondrogenic induction and NP cell formation [91]. It is also associated with fibrosis occurrence and severity in the kidneys [92] and lungs [87] via TGF-β [87] and NAV3-YAP1 signaling [92]. Interestingly, fibrotic matrisome elements appear enriched in the Sox9-expressing region in degenerative NP [25]. Connective tissue growth factor (CTGF, or CCN2) is the putative downstream element of TGF-β1 and can induce the proliferation of active fibroblasts and contribute to tissue fibrosis [93]. CTGF was found to be upregulated in overloaded human NP cells [50] and IVDs [94]. The role of Sox9 and CTGF in NP fibrosis warrants further exploration.

### 4.2. Inflammation

Chronic inflammation is thought to be the major cause of tissue fibrosis via prolonged activation of inflammasomes (e.g., NLRP3) [95,96] and proinflammatory cytokines (e.g., IL-36 and IL-13) [97]. Pro-inflammatory mediators partake in both homeostatic and catabolic regulation of IDD [2,98]. In a chronic inflammatory state, IDD is associated with increased expression of multiple inflammatory cytokines, such as IL-1, IL-6, IL-8 and IL-13, and TNF-α [98]. TNF-α and IL1β are essential to the development of pulmonary fibrosis and are able to accentuate TGF-β-driven EMT or EnMT (endothelial-to-mesenchymal transition) [97]. IL-1β was shown to stimulate the expression of collagen I and III in human NP cells, suggesting a fibroblastic phenotype [60]. IL-36 is a group of cytokines in the IL-1 family and fosters the secretion of profibrotic regulators, leading to fibrosis in the lungs, kidneys, heart, intestines and pancreas [99]. The specific role of IL-36 in NP fibrosis or IDD has never been studied. IL-13 is produced by activated Th2 cells and a crucial fibrogenic mediator in idiopathic pulmonary fibrosis through its receptor IL-13Rα2 [100]. sIL-13Ralpha2-Fc can inhibit IL-13 and decrease collagen I in NP [54] and the impact may possibly be attributed to ADAMTS-8 repression [53]. In contrast, IL-10 is widely reported as an anti-inflammatory and anti-fibrotic cytokine. Injection of IL-10 delayed degenerative changes in a rat tail puncture model via the suppression of p38 MAPK signaling [101], though whether NP fibrosis is targeted is a matter that awaits further investigation. On the other hand, NF-κB is a critical inflammatory component in IDD [102,103] and important to NLRP3 inflammasome activation [104]. Hou et al. showed that NF-κB activation is essential to myofibroblast generation in inflammatory pulmonary fibrosis [73]. Moreover, the NLRP3 inflammasome is associated with inflammation, pyroptosis, ECM degradation and cellular apoptosis in IVD [105]. The NF-κB–NLRP3–caspase-1–IL-1β–IL-18 axis has been shown to form a pathogenic cycle with the TGF-β/Smad signaling pathway to control cardiac fibrosis [106]. It is possible that NLRP3 interacts with TGF-β signaling in regulating NP fibrosis. The roles of different cytokines and inflammasomes in NP fibrosis and IDD have yet to be further elaborated.

### 4.3. Mechanical Load

Mechanical cues in fibrosis progression can be both the cause and result of fibroblast activation [107,108]. Excessive mechanical stress could be one of the major factors in IDD [109] resulting in over-activation of TGF-β [86], YAP/TAZ-mediated cell apoptosis [110] and inflammatory activation, both in vitro [102,104,111] and in vivo [102,112]. This might ultimately cause imbalanced ECM metabolism, rendering NP fibrosis [108,113]. TGF-β signaling is widely reported in mechanical overload-induced fibrosis in the heart [114] and skin [115]. NF-κB is an important inflammatory element in pulmonary fibrosis [73]. Inflammatory NP cells exhibit disrupted F-actin structure and cell volume dysregulation [116]. Cell shape control is crucial to the matrix stiffness-triggered fibroblastic phenotype in NP cells [117]. Upregulation of collagen I, CTGF and α-SMA was observed in NP cells cultured on stiff matrices (41.7 Kpa) [52], under cyclic stretching [50] and in a bipedal model [52]. Increased collagen I was also observed in AF progenitors under cyclic tensile strain [67]. Notably, this process involved nuclear–cytoplasmic shuttling of myocardin-related transcription factor A (MRTF-A) [50,52]. MRTF-A is an important mechanosensing transcriptional coactivator, together with YAP/TAZ, which conveys mechanical stress and contractile cytoskeleton signals to regulate *ACTA2* (coding α-SMA) gene expression. MRTF-A has been shown to translocate to the nucleus and form a complex with serum response factor (SRF) in the promoter region of the CArG regulatory sequence [CC(A/T)6GG] of collagen I, CTGF, α-SMA and other fibrosis-related genes [118]. In a disc puncture model, boundary-constraint disruption and residual-strain loss in the AF caused aberrant mechanosensing, which in turn triggered collagen disorganization and the fibrotic transformation of NP, presumably via MRTF-A and YAP/TAZ [119]. In a bovine disc organ culture, the Hippo-pathway component MST1 was found to respond to high compressive and torsional stresses [120]. TRPC1, TRPV4 and TRPM7 have mechanosensing functions and were found to be expressed in disc cells [108,116]. A compelling role of paratensile signaling in mechanotransdution has recently been suggested [107]. Traction forces could be directed through the matrix fibers and further activate DDR2 and calcium influx for MRTF-A shuffling and myofibroblast generation [121]. Whether the aforementioned mechanosensing machineries are involved in NP fibrosis, particularly in the overload-induced IDD model, warrants further investigation.

### 4.4. Matrix-Degrading Enzymes

The co-occurrence of fibrotic matrix synthesis and matrix metalloproteinase (MMP)-mediated matrix degradation suggests the dynamic nature of fibrogenesis. MMPs that are involved in idiopathic pulmonary fibrosis [122] and hepatic fibrosis [123] have been reviewed recently. These MMPs present multifaceted roles in fibrosis, including cell proliferation, migration and apoptosis beyond degrading collagens. MMP-2 and MMP-1, -11 and -14 are positively correlated with macrophage activation, myofibroblast differentiation and fibrogenesis [122,123]. Conversely, roles for MMP-10 and MMP-13 in fibrosis recovery have been indicated [122,123]. Contrasting roles for MMP-9 were found in both lung and liver fibrosis. MMP3 is essential to pulmonary [124] and myocardial fibrosis [125], and its deficiency dramatically decreases myofibroblast population and inhibits fibrosis. MMP-3 and MMP-7 could proteolytically degrade E-cadherin, resulting in β-catenin liberation and nuclear translocation, thereby driving the expression of fibrotic genes, such as α-SMA and fibronectin, in kidney [126] and lung [124] fibrosis.

Many MMPs (MMP-1, -2, -3, -7, -10 and -13) and ADAMTSs (ADAMTS-1, -4, and -15) that are expressed at low levels or otherwise not detected in healthy IVDs were found to be induced in IDD [127,128]. MMP activity in IDD can be regulated by inflammation, mechanical stress, oxidative stress and fibrogenic cytokines. MMP-2 was observed to dissociate the E-cadherin–β-catenin complex and increase the nuclear translocation and binding of β-catenin with lymphoid enhancer-binding factor 1 (LEF1) for the expression of fibrotic genes, such as α-SMA and collagen I [57]. MiR-29a, which silences MMP-2 expression, could retard NP fibrosis [57]. MMP-12 is critical to myofibroblast induction [129,130,131]. Upregulation of MMP-12 was observed in overload-induced fibroblastic NP cells [50] and in rat injury-induced NP fibrosis [6]. MMP-12 was colocalized with α-SMA in NP cells, suggesting a link with myofibroblast generation [6]. MMP-12 could cleave N-cadherin and further elevate β-catenin activity in smooth muscle cells [132], regulate the bid-activated pathway [131] or the profibrotic genes EGR1 and CYR61 [130] in pulmonary fibrosis, and enhance collagen deposition by limiting the activity of anti-fibrotic extracellular matrix-degrading enzymes MMP-2 and MMP-13 in liver fibrosis [129]. MMP-12 was also identified as a transcription factor that mediates innate immune responses [133]. These findings make manifest the complex regulatory mechanism of MMP-12 in tissue fibrosis. In addition, ADAMTS-8 was found downstream of IL-13 agonist-mediated fibrosis reduction in a rat tail puncture model [53,54]. On the other hand, the high temperature requirement factor A1 (HTRA1) could proteolytically activate latent TGF-β1 in keloid fibroblasts [134]. Studies have showed that HTRA1 was increased in NP fibrosis [21] and that it could produce fibronectin fragments [135], leading to higher levels of IL-6 and -8 [136] and MMP-1, -3 and -13 in IVD cells [135,137]. Intriguingly, HTRA1-generated chondroadherin fragments can distinguish human IDD from normal disc aging [138]. The roles of other MMPs, particularly myofibroblast-related activities, in NP fibrosis await investigation. Whether MMP inhibitors have anti-fibrotic effects and can modify IDD is a question worth examining.

## 5. Approaches to Fibrosis Assessment

IDD is histologically characterized by a dense matrix formation in NP [94] and an NP–AF boundary [52] indiscernible with H&E staining, as well as loss of Safranin-O and an increase in Fast green from Safranin-O–Fast green staining [26,51,139]. Gomori trichrome [36], Masson trichrome [54,57,58,59], picro-sirius red [6,25,26,51,53,55] and FTIR staining [51] have been used to reveal NP fibrosis. Second harmonic generation (SHG) imaging has been deployed to assess the magnitude and pattern of collagen disarrangement in AF [140]. Whether it can provide an effective means to evaluate NP fibrosis awaits determination. Enriched fibrotic matrix components, including biglycan, decorin, fibronectin, and collagen I and III, in addition to reduced aggrecan and collagen II, could be immunodetected. Sophisticated characterization of ECM compositional changes can be profiled by matrisome proteomics [21,25]. Dysregulated collagen fibrillogenesis could be determined by scanning electron microscopy (SEM). Moreover, the immunopositivity of markers for myofibroblasts, including α-SMA, FAP-α and FSP-1, and profibrogenic mediators of TGF-β1, CTGF, MMP-2/-12 and HSP-47, may indicate fibrosis. Nuclear accumulation of β-catenin and the upregulation of components in TGF-βR1-Smad2/3 and RhoA–MRTF-A signaling could be additional indicators.

## 6. Fibrosis and Disc Repair

Cumulative evidence indicates that NP fibrosis is an important feature of IDD. Nevertheless, the impact of fibrosis in disc degeneration and repair is still unclear and the results obtained so far are contradictory (Table 1). For example, MSCs have been reported to induce disc regeneration [26,141] and inhibit NP fibrosis [58], in part via the reduction of fibrogenic mediators MMP-12 [58] and IL-13 [142]. On the other hand, bleomycin could induce fibroblastic transition of NP cells and, when injected into rat tail puncture IVDs, induce fibrosis to maintain disc height despite the progressive loss of T2 signal intensity [26]. Dermal fibroblast implantation in a rat tail disc puncture model could increase collagen I deposition in a dose-dependent manner [56]. Chen et al. claimed that a dermal fibroblast induced NP fibrosis and thus alleviated IDD by maintaining disc height, bending strength and load mechanics, and rescuing T2 signal intensity [55]. However, Shi et al. demonstrated that human neonatal dermal fibroblasts at a low dosage could increase collagen II and reduce fibrosis in a rabbit lumbar disc puncture model [56]. Although NP fibrosis could not be ruled out, enhanced collagen II deposition appeared to contribute to the repair and disc mechanics. Further investigations should verify whether fibrosis has a role in providing a compensatory or tentative protective mechanism for maintaining disc biomechanical function in IDD.

## 7. Conclusions

Findings in clinical specimens and animal models indicate that NP fibrosis is a key feature in IDD. The degradation of disc matrices and the synthesis of fibrotic components, activation of profibrogenic factors, abnormal mechanics and myofibroblast activity are in line with common characteristics of tissue fibrosis. Further understanding NP fibrosis and its regulation may shed light on new strategies for IDD management.

## Figures and Tables

**Table 1:** Studies of fibrosis-related components in IDD and disc cell models. NP: nucleus pulposus; AF: annulus fibrosus; H-dNP: human degenerative NP tissues; hNPCs: human NP cells; AFCs: AF cells; DP: disc puncture surgery; SO–FG: Safranin-O–Fast green staining; PSR: Picro-sirius red staining; MTR: Masson trichrome staining; SEM: scanning electron microscopy; ↑: expression increased; ↓: expression decreased.

Tissue/Cells	Animal Model	Fibrosis Measures	Cellular Morphology	Molecular Markers	Molecular Mechanism	Hallmark of IDD	Reference
H-dNP; overloaded hNPCs	n/a		Spindle	Col1a1 ↑; CTGF ↑; MMP-12 ↑; MRTF-A↑; Acan/Col2a1 ↓	RhoA/MRTF-A signaling		[50]
H-dNP; bleomycin-treated NPCs/AFCs	Mouse Tail DP; bleomycin injection	Histology: SO–FG (FG positivity); PSR		TGF-βR1 ↑; TGF-β ↑; FSP1 ↑; Col1a1 ↑; FN1 ↑; MMP-3/-13 ↑; Col2a1 ↓	TGFβR1-Smad2/3 pathway	N (Fibrosis maintains disc height and stress tolerance)	[26]
H-NPCs on stiff substrate	Rat bipedal model	H&E (indistinct NP–AF boundary)	Spindle	Col1a1 ↑; CTGF ↑; α-SMA ↑;	MRTF-A nuclear translocation	Y	[52]
n/a	Rat tail DP; IL13 agonist injection	PSR	n/a	Collagen I ↑;	IL-13 agonist reduced ADAMTS-8	Y	[53]
n/a	Rat tail DP; IL13 agonist injection	MTR	n/a	Collagen I ↑; collagen II ↓	IL-13 agonist reduced fibrosis	Y	[54]
IL1-treated H-NPCs	n/a	n/a		Collagen I ↑; collagen III ↑	TGF-β aggravated fibrosis via ANGPTL2	Y (Fibrosis related to disc inflammation)	[60]
Rat NPCs coculture with fibroblasts	Rat tail and cynomolgus monkey lumbar DP; dermal fibroblast injection	PSR	n/a	FSP-1 ↑; collagen I ↑	TGF-βR1-Smad2/3 pathway	N (Fibrosis maintains disc height and compressing and bending tolerance)	[55]
n/a	Mouse tail DP	n/a	Fibroblast-like cells	FSP-1 ↑; α-SMA ↑; Col1a1 ↑; FAP-α ↑	n/a	Y	[49]
n/a	Rabbit lumbar DP; neonatal human or rabbit dermal fibroblast injection	n/a	n/a	Collagen I ↑	n/a	Y (Higher Collagen II/I ratio indicates repairing strength)	[56]
n/a	Aging IDD; TonEBP deficiency	SO–FG (FG positivity); PSR; FTIR	Honeycomb chondrocyte-like	Collagens ↑; lamellar disorganization	n/a	Y	[51]
n/a	SM/J mice	Matrisome proteomics	Chondrocyte-like	Col18a1 ↑; Col6a1/a2 ↑; Bgn ↑; Dcn ↑; Vcan ↑; Prelp ↑; Fn1 ↑; Comp ↑	n/a	Y	[25]
n/a	HIF deficiency mice	SO–FG (FG positivity)		n/a	HIF deficiency developed fibrosis	Y	[139]
Rabbit NPCs	Rabbit lumbar DP; microRNA-29 local delivery	MTR	Stress fibers	α-SMA ↑; collagen I ↑	MMP2-mediated activation of β-catenin	Y	[57]
TGF-β1-treated H-NPCs	Rat tail DP; NR4A1 overexpression	Gross appearance; MTR	Stress fibers	α-SMA ↑; Col1a1 I ↑; SERPINE1 ↑; SMAD7 ↑	NR4A1 bound with SP1 to repress TGF-β-targeted genes	Y	[59]
H-dNP		Proteomics		Collagen I ↑; biglycan ↑; decorin ↑; Prelp ↑; fibronectin ↑; CILP ↑	n/a	Y (Fibrosis related to aging and IDD)	[21]
H-NP and NPCs	Rat/mouse tail DP	PSR	Myofibroblast-like	α-SMA ↑; Col1a1 I ↑; FSP-1 ↑; FAP-α ↑; MMP-12 ↑	n/a	Y	[6]
n/a	Rabbit lumbar DP; MSC injection	MTR; SEM	n/a	Collagen I ↑; MMP-12 ↑; HSP-47 ↑; collagen fibrillogenesis	n/a	Y	[58]
Mechanical stress on H-NPCs	Rat tail DP	Gomori trichrome	n/a	MMP-2 ↑; Col1a1 ↑; periostin ↑; Sox9 ↓	n/a	Y	[36]
H-NP	n/a	H&E (dense matrix)	Spindle	CTGF ↑	n/a	Y	[94]

## Data Availability

All data included in this manuscript are from published scientific manuscripts and available on PubMed (NIH).
